# Phlorotannins from *Ecklonia cava* Attenuates Palmitate-Induced Endoplasmic Reticulum Stress and Leptin Resistance in Hypothalamic Neurons

**DOI:** 10.3390/md17100570

**Published:** 2019-10-09

**Authors:** Seyeon Oh, Myeongjoo Son, Junwon Choi, Chang Hu Choi, Kook Yang Park, Kuk Hui Son, Kyunghee Byun

**Affiliations:** 1 Functional Cellular Networks Laboratory, College of Medicine, Department of Medicine, Graduate School and Lee Gil Ya Cancer and Diabetes Institute, Gachon University, Incheon 21999, Korea; seyeon8965@gachon.ac.kr (S.O.); mjson@gachon.ac.kr (M.S.); choijw88@gc.gachon.ac.kr (J.C.); 2 Department of Anatomy & Cell Biology, Gachon University College of Medicine, Incheon 21936, Korea; 3 Department of Thoracic and Cardiovascular Surgery, Gachon University Gil Medical Center, Gachon University, Incheon 21565, Korea; cch624@gilhospital.com (C.H.C.); kkyypark@gachon.ac.kr (K.Y.P.)

**Keywords:** obesity, *Ecklonia cava*, phlorotannins, hypothalamic neurons, microglia, toll-like receptor 4, endoplasmic reticulum stress, leptin resistance

## Abstract

Leptin resistance in the hypothalamus has an essential role in obesity. Saturated fatty acids such as palmitate bind to Toll-like receptor 4 (TLR4) and lead to endoplasmic reticulum (ER) stress and leptin resistance. In this study, we evaluated whether extracts of *Ecklonia cava* would attenuate the ER stress induced by palmitate and reduce leptin resistance in hypothalamic neurons and microglia. We added palmitate to these cells to mimic the environment induced by high-fat diet in the hypothalamus and evaluated which of the *E. cava* phlorotannins—dieckol (DK), 2,7-phloroglucinol-6,6-bieckol (PHB), pyrogallol-phloroglucinol-6,6-bieckol (PPB), or phlorofucofuroeckol-A (PFFA)—had the most potent effect on attenuating leptin resistance. TLR4 and NF-κB expression induced by palmitate was attenuated most effectively by PPB in both hypothalamic neurons and microglia. ER stress markers were increased by palmitate and were attenuated by PPB in both hypothalamic neurons and microglia. Leptin resistance, which was evaluated as an increase in SOCS3 and a decrease in STAT3 with leptin receptor expression, was increased by palmitate and was decreased by PPB in hypothalamic neurons. The culture medium from palmitate-treated microglia increased leptin resistance in hypothalamic neurons and this resistance was attenuated by PPB. In conclusion, PPB attenuated leptin resistance by decreasing ER stress in both hypothalamic neurons and microglia.

## 1. Introduction

Leptin resistance is a major pathophysiology of obesity. Leptin plays a crucial role in the control of body weight and in regulating food intake by binding to the long isoform of leptin receptor (ObR) in the hypothalamic nuclei [1,2]. However, the action of leptin is hindered in diet-induced obese mice or obese humans who have high serum leptin level; this phenomenon is called leptin resistance. Leptin resistance is caused by impaired leptin signaling in spite of high serum level of leptin [3,4]. Reduction of the activity of the Janus-activating kinase2-signal transducer and activator of transcription 3 (JAK2-STAT3) signaling pathway is one cause of leptin resistance [5,6,7].

Endoplasmic reticulum (ER) stress has been proposed to be the predominant cause of leptin resistance [8]. ER stress activates the unfolded protein response (UPR), which decreases ER stress. UPR includes a pathway that involves inositol-requiring enzyme 1 (IRE1), PKR-like ER kinase (PERK), and activating transcription factor 6 [9]. A failure of the UPR to attenuate ER stress leads to apoptosis [9]. It is well known that saturated fatty acids (SFA) such as palmitate induce ER stress by binding to Toll-like receptor 4 (TLR4) in hypothalamic neurons and lead to leptin resistance [10,11,12]. Palmitate activates not only hypothalamic neurons but also microglia via the TLR4 pathway, and microglia activation leads to neuronal cell death [13].

*Ecklonia cava* is a brown alga that contains two major groups of possibly functional components: polyphenols and polysaccharides [14]. Unique polyphenolic phlorotannins are a large class of well-characterized marine secondary metabolites. The phlorotannins from the *E. cava* have multiple biological activities such as anti-inflammatory [14,15], antioxidant [16], and antiadipogenic activities [17,18,19,20]. Phlorotannins (e.g., eckol, dieckol, and phlorofucofuroeckol A) have anti-obesity effects in zebrafish [17], mice [18,19,20], and cell cultures [21]. However, those studies evaluated anti-obesity effects mainly on the basis of the improvement in lipid metabolism such as inhibition of adipogenesis or lipid accumulation [17,19]. Although leptin resistance in the brain has an essential role in obesity, the effect of *E. cava* extract on leptin resistance has not been revealed. Here, we evaluated whether extracts of *E. cava* would attenuate the ER stress induced by palmitate and decrease leptin resistance in hypothalamic neurons and microglia. In addition, we compared the leptin resistance–attenuating effect of 4 phlorotannin—dieckol (DK), 2,7-phloroglucinol-6,6-bieckol (PHB), pyrogallol phloroglucinol-6,6-bieckol (PPB), or phlorofucofuroeckol A (PFFA).

## 2. Results and Discussion

### 2.1. The Attenuating Effects of E. cava Extract on TLR4 Expression and Cell Death in Palmitate-Treated Hypothalamic Neurons and Microglia

It has been suggested that high-fat diet (HFD) increases the levels of plasma free fatty acids (FFAs), which raises the FFA levels in the hypothalamus, the main center of weight maintenance. HFD, which is rich in the SFA palmitate, specifically increases palmitate levels in the hypothalamus [22]. Neurons may directly sense FFAs by TLR4 [23].

**Figure 1:**
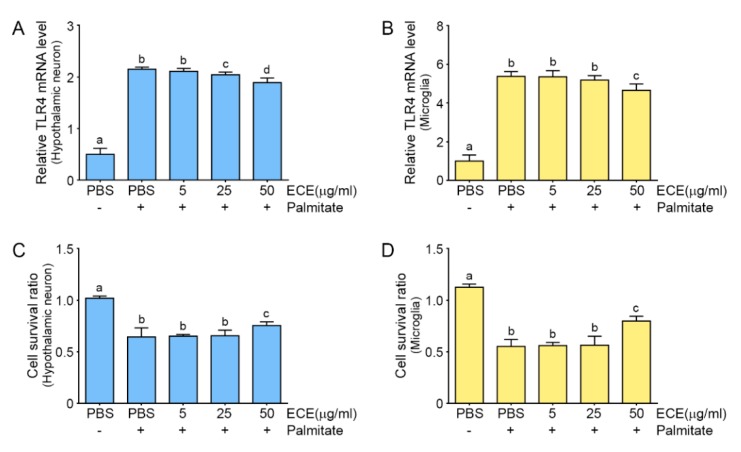
Effects of *E. cava* extract on TLR4 expression and cell death in palmitate-treated hypothalamic neurons and microglia. The hypothalamic neurons and microglia were exposed to palmitate in phosphate buffered saline (PBS) or in different concentrations of *E. cava* extract (ECE). (**A**) In hypothalamic neurons, TLR4 mRNA levels were increased by palmitate in PBS. Addition of ECE decreased TLR4 mRNA levels in a concentration-dependent manner, with significant effects at 25 μg/mL and 50 μg/mL ECE. (**B**) In microglia, TLR4 expression was increased by palmitate, but was significantly decreased at 50 μg/mL ECE. (**C**,**D**) The survival ratios of hypothalamic neurons and microglia were measured using a cell survival assay after incubation with palmitate in PBS or different concentrations of ECE. Cell survival ratio was decreased by palmitate and was significantly increased by ECE at 50 μg/mL. Data are means ± SD. Values with different letters are significantly different at *p* < 0.05. TLR4, Toll-like receptor 4.

We added palmitate to hypothalamic neurons and microglia to mimic the environment induced by HFD in the hypothalamus. SFAs directly and quickly trigger inflammation in microglia [24], suggesting that both neurons and microglia are related to the changes in the hypothalamus induced by HFD. First, we evaluated the effect of different concentrations of *E. cava* extract (ECE) on TLR4 expression in palmitate-treated hypothalamic neurons and microglia. The expression of TLR4 was increased by palmitate in both cell types. When ECE was added, the TLR4 expression was decreased by ECE in a concentration-dependent manner (Figure 1A,B; Figure S2A,B). The TLR4 expression level was significantly lower at 50 μg/mL than at 25 μg/mL; however, it was not significantly different among 50, 100, and 200 μg/mL. The cell survival ratio of both cell type was significantly higher at 50 μg/mL than 25 μg/mL; however, it was not significantly different among 50, 100, and 200 μg/mL. (Figure 1C,D; Figure S2C,D). Among dosages of ECE which we tested, decreasing TLR4 expression and cell death was significantly reversing at 50 μg/mL of ECE concentration. From these results, we calculated the exact amount of 4 phlorotannins to be added to both cell types. *E. cava* extract contains 3.57% (w/w) PPB, which is the least of the 4 phlorotannins. So, 50 μg/mL *E. cava* extract was expected to contain 1.8 μg/mL PPB [25]. To match this dosage, we treated both cells types with 1.8 µg/mL phlorotannins (DK, PHB, PPB, or PFFA).

### 2.2. Phlorotannins Attenuated TLR4 Expression Induced by Palmitate in the Hypothalamic Neuron and Microglia

**Figure 2:**
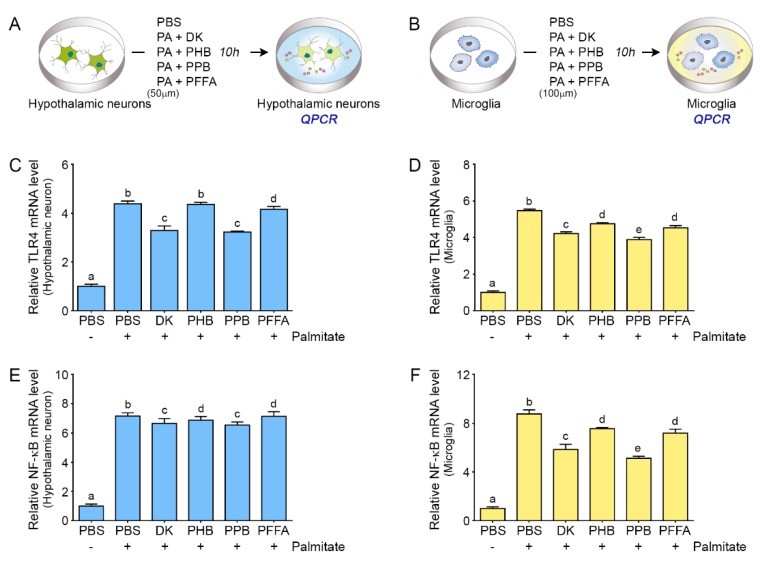
Effects of phlorotannins on TLR4 and NF-κB expression in palmitate-treated hypothalamic neurons and microglia (**A**,**B**) Schematic illustration of the treatment. Hypothalamic neurons and microglia were treated not only with palmitate but also with phlorotannins. (**C**) In hypothalamic neurons, the TLR4 mRNA level increased by palmitate treatment was decreased significantly by DK and PPB. (**D**) In microglia, the TLR4 mRNA level increased by palmitate was decreased significantly by all 4 phlorotannins; PPB had the most significant effect. (**E**) In hypothalamic neurons, the mRNA level of NF-κB was increased by palmitate treatment and was significantly decreased when palmitate was supplemented with DK or PPB. (**F**) In microglia, the mRNA level of NF-κB was increased by palmitate and was significantly decreased when palmitate was supplemented with any of the 4 phlorotannins; the strongest effect was observed with PPB. Data are means ± SD. Values with different letters are significantly different at *p* < 0.05. TLR4, Toll-like receptor 4; NF-κB, nuclear factor kappa-light-chain-enhancer of activated B; PBS, phosphate-buffered saline; DK, dieckol; PHB, 2,7-phloroglucinol-6,6-bieckol; PPB, pyrogallol-phloroglucinol-6,6-bieckol; PFFA, phlorofucofuroeckol A.

TLR4 expression induced by palmitate was attenuated by all 4 phlorotannins tested in both hypothalamic neurons and microglia (Figure 2A,D); in hypothalamic neurons, PPB and DK of 1.8 μg/mL significantly attenuated TLR4 expression than PBH or PFFA (PPB = DK > PFFA > PHB). In case of microglia, PPB showed most significant deceasing of TLR4 expression than 3 other phlorotannins (PPB > DK > PHB = PFFA). In the hypothalamus, TLR4 is predominantly expressed by microglia [12]. During chronic HFD feeding, hypothalamic TLR4 expression and activity are increased [26,27]. TLR4 signaling is involved in the development of ER stress [28].

### 2.3. Phlorotannins Induced Decreasing NF-κB Expression Induced by Palmitate

TLR4 activation by SFAs results in the activation of nuclear factor kappa-light-chain-enhancer of activated B cells (NF-κB) and leads to ER stress [29]. In our study, the expression of NF-κB was increased by palmitate treatment in both hypothalamic neurons and microglia, and the effect of palmitate was decreased by adding ECEs at a concentration of 1.8 μg/mL. The attenuating effect on NF-κB in the hypothalamic cells were higher by PPB and DK than by PHB or PFFA (PPB = DK > PHB = PFFA). Among the 4 phlorotannins, PPB showed the most significant attenuation effect on NF-κB expression increased by palmitate in microglia (PPB > DK > PHB = PFFA) (Figure 2E,F). DK and PPB have specific phenol structure, which has hydroxy groups at the 1,3,5 sites of benzenetriol base and they are structural derivatives of phloroglucinol. Due to the specificity of the chemical structure, DK and PPB have known to play a radical scavenger role. Tyrosinase and ligand–enzyme interaction analysis of DK, which structure is considerably well known compared to PPB, indicate that it has an active site at His208, Met215, and Gly4. Also, as DK has high binding energy (−126.12 kcal/mol) according to results of docking experiments, it seems that it might play an important role in receptor–ligand interaction [30]. The existence of an electron donor in the hydroxyl group and phenyl ring of phenolic compounds might also be expected to provide these results. Thus, even though there is no direct experimental evidence of PPB, we could expect similar results from PPB as those of DK since it has more than 10 hydroxyl groups [31,32].

### 2.4. Phlorotannins Attenuated ER Stress Induced by Palmitate

**Figure 3:**
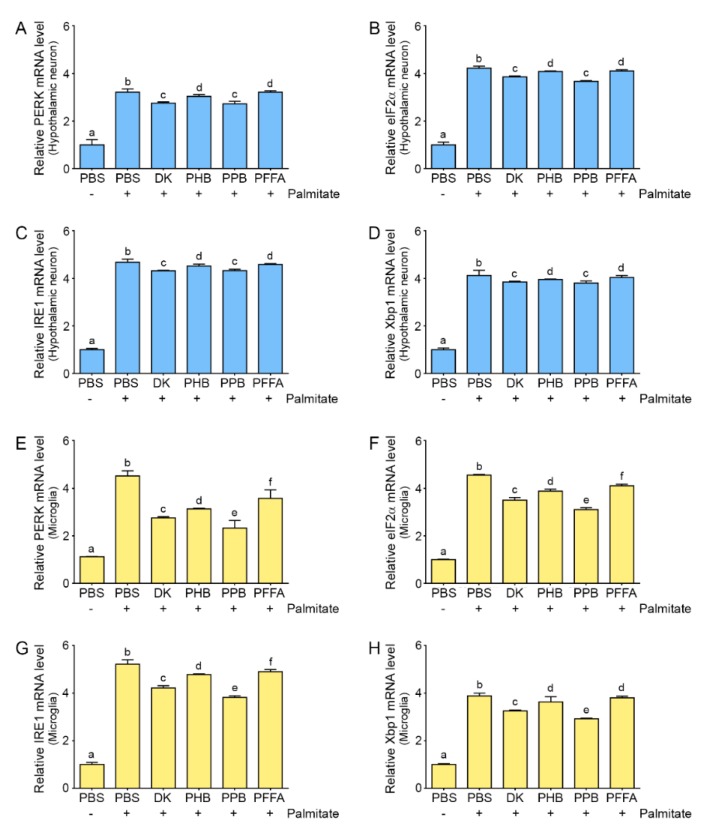
Effects of phlorotannins on ER stress in palmitate-treated hypothalamic neurons and microglia. (**A**–**D**) In hypothalamic neurons, the mRNA levels of the ER stress markers (PERK, eIF2α, IRE1, and Xbp1) were significantly increased by palmitate and were significantly decreased when palmitate was supplemented with any of the 4 phlorotannins—DK and PPB had the most significant effects. (**E**–**H**) In microglia, the mRNA levels of the same markers were significantly increased by palmitate and were significantly decreased when palmitate was supplemented with any of the 4 phlorotannins—PPB had the most significant effect. Data are as means ± SD. Values with different letters are significantly different at *p* < 0.05. ER, endoplasmic reticulum; ECE, *E. cava* extract; PERK, PKR-like ER protein kinase; eIF2α, eukaryotic initiation factor 2 alpha; IRE1, inositol-requiring-enzyme-1; Xbp1, X-box-binding protein 1; PBS, phosphate-buffered saline; DK, dieckol; PHB, 2,7-phloroglucinol-6,6-bieckol; PPB, pyrogallol-phloroglucinol-6,6-bieckol; PFFA, phlorofucofuroeckol A.

Expression of the ER stress markers, PERK, eukaryotic initiation factor 2 alpha (eIF2α), IRE1, and X-box-binding protein 1 (Xbp1), in both hypothalamic neurons and microglia was increased by palmitate (Figure 3). The expression of these markers increased by palmitate was decreased by ECEs in the hypothalamic neuron, and the attenuation effect was higher by adding DK and PPB than PHB or PFFA (PPB = DK > PHB = PFFA). The enhanced expression of ER stress markers in the microglia by palmitate was decreased most significantly by adding PPB (PPB > DK > PHB = PFFA). IRE1 and PERK phosphorylation, and XBP1 splicing increase in the hypothalamus of HFD-induced obese rodents [29,33,34]. In various in vitro models that used hypothalamic neuronal cell lines such as Agrp-expressing mHypoE-42 (N42) [35] or mHypoE-44 neurons [10], palmitate induces ER stress.

The role of the UPR or ER stress in non-neuronal cells has been less studied than in neurons. TLR4 signaling activated by SFAs activates the UPR and induces the IRE1–XBP1 axis in macrophages [36]. Deletion of IRE1a from cells of the myeloid lineage such as macrophages has a protective effect against HFD-induced obesity [37]. In the rodent and human central nervous system, XBP1 is expressed in the microglia [38,39]. In both hypothalamic neurons and microglia, which express TLR4, ER stress seems to be induced by palmitate, and TLR4 and NF-κB are involved in ER stress induction. In our study, ER stress was induced by palmitate in both hypothalamic neurons and microglia, and TLR4 mRNA levels were increased by palmitate in both cell types. ER stress in both cell types was decreased by phlorotannins (Figure 3).

### 2.5. PPB is Most Efficient in Attenuating Leptin Resistance in Palmitate-Treated Hypothalamic Neurons

The activation of inhibitor of NF-κ-B kinase subunit beta (IKKβ)/NF-κB signaling increases the level of suppressor of cytokine signaling 3 (SOCS3) [29]. SOCS3 expression is a potent negative regulator of the JAK-STAT3 pathway and leads to leptin resistance [40,41]. SOCS3 subsequently decreases ObR expression by inhibiting JAK-STAT3 activation [42].

**Figure 4:**
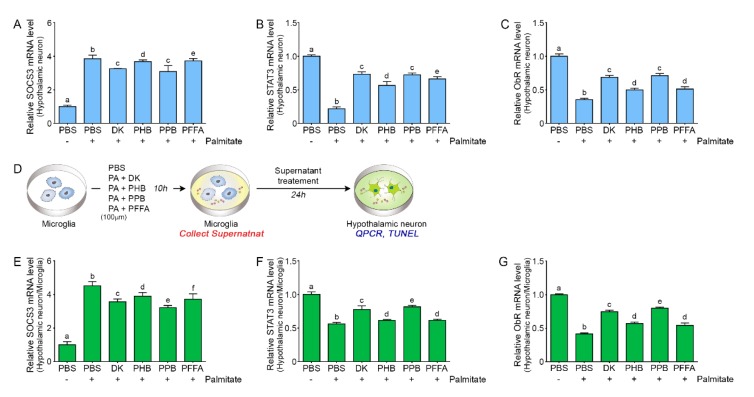
Effects of phlorotannins on the expression of factors involved in leptin resistance in hypothalamic neurons and the effect of microglia (**A**) Expression of SOCS3 in hypothalamic neurons was increased by palmitate. The SOCS3 mRNA level increased by palmitate was significantly decreased by the 4 phlorotannins; DK and PPB had the most significant effects. (**B**,**C**) STAT3 and ObR mRNA levels were decreased by palmitate. In the presence of palmitate, they were significantly increased by the 4 phlorotannins; DK and PPB had the most significant effects. (**D**) Illustration explaining processes of how to make cell samples. Microglia was treated not only with palmitate but also with phlorotannins and then, the microglia supernatant was incubated with hypothalamic neurons. (**E**) In hypothalamic neurons incubated with microglial supernatant, SOCS3 expression was increased by supernatant from microglia treated with palmitate. The SOCS3 mRNA level increased by palmitate was significantly decreased by the 4 phlorotannins; PPB had the most significant effect. (**F**,**G**) In hypothalamic neurons treated with microglial supernatant, STAT3 and ObR expression was significantly decreased by palmitate. In the presence of palmitate, it was significantly increased by phlorotannins; PPB had the most significant effect. Data are means ± SD. Values with different letters are significantly different at *p* < 0.05. SOCS3, suppressor of cytokine signaling 3; ObR, leptin receptor; PBS, phosphate-buffered saline; DK, dieckol; PHB, 2,7-phloroglucinol-6,6-bieckol; PPB, pyrogallol-phloroglucinol-6,6-bieckol; PFFA, phlorofucofuroeckol A.

In our study, the expression of SOCS3 was increased by palmitate in hypothalamic neurons, and this increase was attenuated by ECE. PPB and DK showed the most potent attenuation effects. STAT3 and ObR expression was decreased by palmitate in hypothalamic neurons, and this decrease was attenuated by ECE; PBB and DK had the strongest effects among the 4 phlorotannins (Figure 4A,C). To evaluate the role of microglia in the pathophysiology of leptin resistance, palmitate was prepared with or without phlorotannin-treated microglia supernatant and hypothalamic neurons were incubated with microglia supernatant (Figure 4D).

Addition of this supernatant to hypothalamic neuron increased SOCS3 expression (Figure 4E). This effect of palmitate was attenuated by phlorotannins, of which PPB had the most significant effect (Figure 4E). Addition of the microglial supernatant to hypothalamic neurons decreased the expression of STAT3 and ObR, and this effect was attenuated by phlorotannins, most significantly by PPB (Figure 4F,G). These results show that the supernatant of palmitate-treated microglia induced leptin resistance in hypothalamic neuron cells and suggest that microglia could be involved directly or indirectly in the development of leptin resistance in hypothalamic neurons. It is known that IKKβ/NF-κB signaling may act both upstream and downstream of ER stress in DIO mice [29,43].

**Figure 5:**
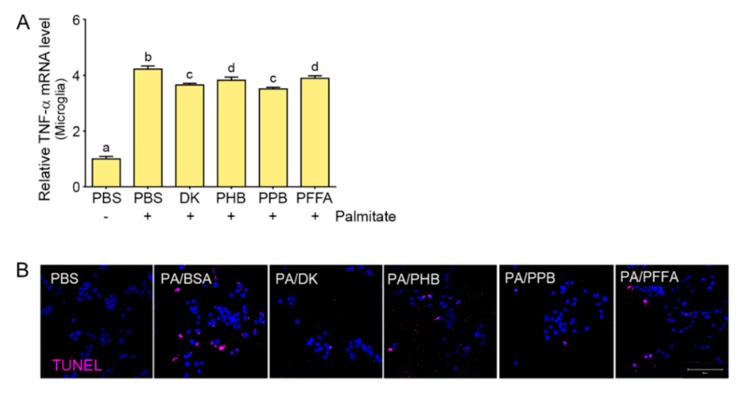
The attenuating effects of PPB on cell death in hypothalamic neuron cell affected by microglia. (**A**) TNF-α expression in microglia was increased by palmitate, and the effect of palmitate was decreased by phlorotannins, most strongly by PPB and DK. Data are means ± SD. Values with different letters are significantly different at *p* < 0.05. (**B**) The design of the experiment was as in Figure 4D. TUNEL stain showing that microglia supernatant incubated apoptotic hypothalamic neurons (pink) were increased by palmitate and decreased by PPB. All cell nuclei were detected by DAPI (blue). TNF-**α**, tumor necrosis factor alpha; TUNEL, terminal deoxynucleotidyl transferase dUTP nick end labeling; PBS, phosphate-buffered saline; DK, dieckol; PHB, 2,7-phloroglucinol-6,6-bieckol; PPB, pyrogallol-phloroglucinol-6,6-bieckol; PFFA, phlorofucofuroeckol A.

The TLR4-NF-κB pathway elevates the levels of TNF-α [38]. TLR4 expression, increased ER stress, and increased TNF-α secretion are involved in leptin resistance development through further activation of the JNK/NF-κB signaling pathways. Furthermore, central nervous system (CNS) exposure to low-dose TNF-α promotes leptin resistance [44]. In our study, TNF-α expression was increased by palmitate and this effect of palmitate was decreased by PPB (Figure 5A). Microglia activated by SFAs via TLR4 release TNF-α, which leads to neuronal cell death [13] and aggravates neuro-inflammation and leptin resistance. In our study, the supernatant from microglia treated with palmitate induced hypothalamic neuron cell death, and PPB and DK showed greater attenuation effect than PHB or PFFA (Figure 5B).

**Figure 6:**
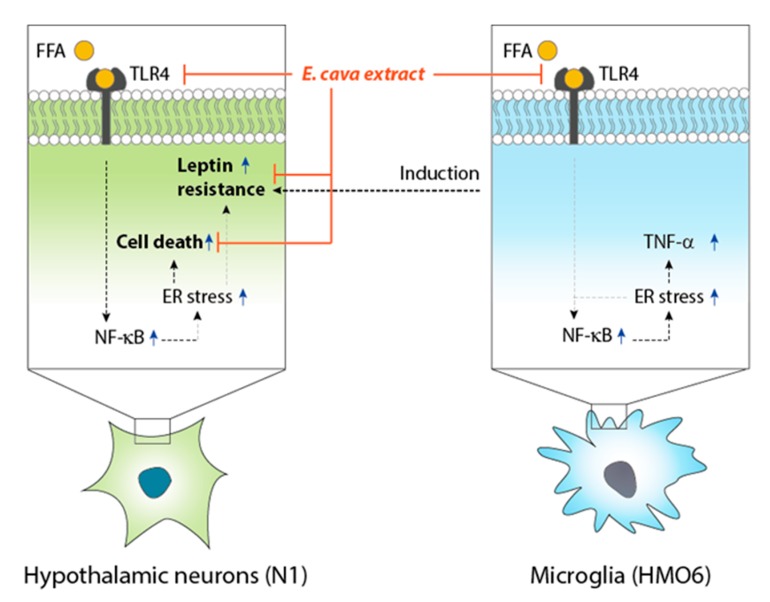
Summary of the findings of this study. Palmitate induced ER stress in hypothalamic neurons via binding to TLR4 and an increase in NF-κB expression. The increased NF-κB and ER stress lead to leptin resistance in hypothalamic neurons, whereas PPB decreased ER stress and leptin resistance. ER stress and NF-κB expression were also increased by palmitate in microglia, leading to an increase in TNF-α expression. TNF-α might induce leptin resistance in hypothalamic neurons. PPB attenuated ER stress, NF-κB expression, and TNF-α expression in microglia and decreased leptin resistance in hypothalamic neurons. ER, endoplasmic reticulum; TLR4, toll-like receptor 4; NF-κB, nuclear factor kappa-light chain enhancer of activated B cells; TNF-α, tumor necrosis factor alpha; ECE, *E. cava* extract; DK, dieckol; PHB, 2,7-phloroglucinol-6,6-bieckol; PPB, pyrogallol-phloroglucinol-6,6-bieckol; PFFA, phlorofucofuroeckol A.

Our study demonstrated that palmitate induced ER stress in hypothalamic neurons via binding to TLR4, and there was an increase in NF-κB activation. The increased NF-κB and ER stress lead to leptin resistance in hypothalamic neurons, whereas phlorotannins decreased ER stress and leptin resistance. ER stress and NF-κB expression were also increased by palmitate in microglia, leading to an increase in TNF-α expression. TNF-α might induce leptin resistance in hypothalamic neurons. Phlorotannins attenuated ER stress, NF-κB expression, and TNF-α expression in microglia and decreased leptin resistance in hypothalamic neurons (Figure 6).

Normalization of leptin response in the hypothalamus or resolving leptin resistance is essential to control obesity or obesity-related diseases. Thus, it seems that the phlorotannins has a therapeutic potential to treat obesity by decreasing leptin resistance in the hypothalamus. However, phlorotannins are required to cross the blood–brain barrier (BBB) to act as agents to control leptin resistance in the hypothalamus. It is generally known that the prediction whether molecules passively cross the BBB or not is possible by Lipinski’s rules. For crossing BBB, the molecular weight has to be less than 500 Da, log *P_oct_* in the range of 2–4, and the number of hydrogen bond donors to be less than 5 [45]. According to Lipinski’s rules, the phlorotannins which we used this study mismatched. However, one study showed that DK crossed BBB and accumulated in the brain. It suggested that DK may be transported via unknown mechanisms, even though it has molecular weights over 700 and a number of polar groups (Figure S3) [46]. 

In our study, DK showed similar effect with PPB for attenuating ER stress and leptin resistance. Thus, DK which might cross the BBB could be an effective material to treat obesity by decreasing leptin resistance in the brain.

## 3. Materials and methods

### 3.1. E. cava Extraction and 4 Phlorotannins Preparation

*E. cava* extraction and 4 phlorotannins preparation methods were described in Supplementary Materials and Methods and this method followed from previous study [24].

### 3.2. Cell Culture and Experimental Cell Models

#### 3.2.1. Cell Culture

Hypothalamic neuron cell line (N1) were purchased from the American Type Culture Collection (ATCC, Manassas, VA, USA). Microglia cell line (HMO6) were provided by Dr Seung U. Kim, Division of Neurology, Department of Medicine, University of British Columbia, Vancouver, BC, Canada [47]. These cells were cultured with high-glucose DMEM, 10% fetal bovine serum, and 1% penicillin/streptomycin at 37 °C under 5% CO_2_.

#### 3.2.2. Optimization of the Experimental Cell Model

To determine the optimal concentration of palmitate for the induction of ER stress, hypothalamic neurons and microglia were treated with 50 to 400 μM palmitate based on previous studies [10,44] and the TLR4 expression was increased from 50 µM palmitate in hypothalamic neurons and from 100 µM palmitate in microglia (Figure S1A,B). Thus, we decided to treat hypothalamic neurons with 50 μM palmitate and microglia with 100 μM palmitate. To determine the time of treatment needed to trigger ER stress, TLR4 expression was examined after treatment of each cell line with palmitate for 10 to 48 h [10,48]. In both cell lines, the mRNA level of TLR4 was increased from 10 h after palmitate addition and treatment time is 10 h in all experiments (Figure S1C,D).

#### 3.2.3. Treatment with *E. cava* Extract

In a previous study [45], we treated endothelial cells with *E. cava* extract at 5, 25, or 50 µg/mL for 10 h and we found the most dramatic effect on cell survival at 50 µg/mL. Therefore, in this study, we treated hypothalamic neurons and microglia with 50 µg/mL of *E. cava* extract for 10 h.

#### 3.2.4. Treatment with 4 Phlorotannins from *E. cava* Extract

*E. cava* extract contains 3.57% (w/w) PPB, so 50 µg/mL *E. cava* extract was expected to contain 1.8 µg/mL PPB [24]. To match this dosage, we treated cells with 1.8 µg/mL phlorotannins (DK, PHB, PPB, or PFFA) for 10 h.

#### 3.2.5. Experimental Cell Model to Determine the Role of Microglia in Leptin Resistance

To validate the role of microglia in leptin resistance, microglia were treated with palmitate (100 μM) in the presence or absence of 4 phlorotannins for 10 h. The microglia supernatants were collected and used to incubate hypothalamic neurons for 1 day. Protein and RNA samples were extracted from the hypothalamic neurons.

### 3.3. Cell Viability Measurement

Hypothalamic neurons and microglia were cultivated in a 96-well plate (10^4^ cells//well) for 24 h and washed with phosphate-buffered saline (PBS). Water-soluble tetrazolium salt solution was added (100 μL/well) for 4 h at 37 °C. Absorbance at 570 nm was measured using a VERSAmax tunable microplate reader (Molecular Devices, San jose, CA, USA).

### 3.4. Sample Preparation

#### 3.4.1. RNA Extraction and cDNA Synthesis

Total RNA was extracted using RNAiso Plus (Takara, 9108, Kusatsu, Shiga, Japan) according to the manufacturer’s instructions. RNAiso Plus (0.5 mL) was mixed with chloroform (0.1 mL) and incubated at room temperature for 7 min. The mixture was centrifuged for 15 min at 4 °C at 12,000× *g*. The supernatant was collected in a new tube, mixed with 0.25 mL of 100% isopropanol, gently shaken and then centrifuged again to precipitate the RNA. The supernatant was discarded and the RNA pellet was washed with 70% ethanol and centrifuged at 7500× *g* for 5 min at 4 °C. The dried pellet was dissolved in 30 μL of diethyl pyrocarbonate water and RNA was quantified using Nano Drop 2000 (Thermo Fisher Scientific, Waltham, M.A., USA). Total RNA (1 µg) was used to synthesize cDNA using the cDNA synthesis kit (TAKARA, 6210A, Kusatsu, Shiga, Japan).

#### 3.4.2. Protein Isolation

Protein from cells treated with palmitate and the 4 phlorotannins of ECE was isolated by adding 500 µL EzRIPA buffer (ATTO, WSE-74210, Amherst, NY, USA) at 4 °C. Sonication was carried out for a total of 5 min with a sonication time of 10 s and a resting time of 1 min. After sonication, the supernatant was harvested after centrifugation at 12,000× *g* for 20 min at 4 °C. The protein content was quantified using BCA assay (Thermo Fisher Scientific, Waltham, MA, USA) and used in experiments.

### 3.5. Real-Time Reverse Transcription Polymerase Chain Reaction (qRT-PCR)

qRT-PCR was performed to analyze the mRNA levels of ER-stress- and leptin-resistance-related factors. The reaction mixtures were prepared in wells of 384-well plates and contained 0.8 µL 10 pM primer (Table S1), 1 µg cDNA template (2 µL), and 5 µL SYBR Green (TAKARA, RR420L, Kusatsu, Shiga, Japan). Analysis was performed using by CFX384 Touch (Bio-Rad, Hercules, CA, USA).

### 3.6. Terminal Deoxynucleotidyl Transferase (TdT)-Mediated dUTP Nick End Labeling (TUNEL)

TUNEL (Transgen Biotech Co., FA201, Haidian, Beijing, China) was used to determine apoptosis in hypothalamic neurons. Cells were cultured in an 8-well chamber at 3 × 10^5^ cells/well and treated with palmitate in the presence or absence of 4 phlorotannins for 10 h. The cells were then fixed at room temperature for 30 min with 4% paraformaldehyde and washed with PBS. For permeabilization, the cells were treated with 0.1% Triton X-100 and 0.1% sodium citrate in PBS at room temperature for 5 min and washed with PBS; 50 µL of labeling solution and 2 µL of TdT in TUNEL kit were mixed and applied to the cells at 37 °C in the dark for 1 h. Cells were washed with PBS and incubated with 4′6-diamino-2-penilinole (DAPI, Sigma-Aldrich, D3286, St Luis, MO, USA) at room temperature for 5 min to stain nuclei. Images were taken under a confocal microscope (Zeiss, LSM 710, Oberkochen, Germany) and analyzed with Zen 2009 software (Zeiss, Oberkochen, Germany).

### 3.7. Statistical Analysis

The statistical techniques used in this study compared the statistical difference among 6 groups using the non-parametric Kruskal–Wallis test, and the difference between 2 groups was compared using the Mann–Whitney U test for post-test. Experiments were performed in triplicate, and results are presented as means ± SD. The analysis was conducted using SPSS version 22 (IBM Co., Armonk, NY, USA).

## 4. Conclusions

In conclusion, phlorotannins attenuated leptin resistance by decreasing ER stress in both hypothalamic neurons and microglia. Especially, DK known to cross BBB could be a therapeutic agent for obesity by decreasing leptin resistance.

## Supplementary Materials

The following are available online at https://www.mdpi.com/1660-3397/17/10/570/s1, Figure S1: Concentration and duration of palmitate causing endoplasmic reticulum (ER) stress in hypothalamic neurons and microglia; Figure S2: Effects of *E. cava* extract on Toll-like receptor 4 (TLR4) expression and cell death in palmitate-treated hypothalamic neurons and microglia; Figure S3. Chemical characteristics of 4 phlorotannins from Ecklonia cava extract; Table S1: List of primer for quantitative real time polymerase chain reaction (qRT-PCR).
