# Supplementary material for: Phlorotannins from Ecklonia cava Attenuates Palmitate-Induced Endoplasmic Reticulum Stress and Leptin Resistance in Hypothalamic Neurons
Source: Mar Drugs. 2019 Oct 9;17(10):570. doi: 10.3390/md17100570 (PMC6835517; doi:10.3390/md17100570)
Supplement: Supplementary file 1 [file marinedrugs-17-00570-s001.pdf]

Supplementary material

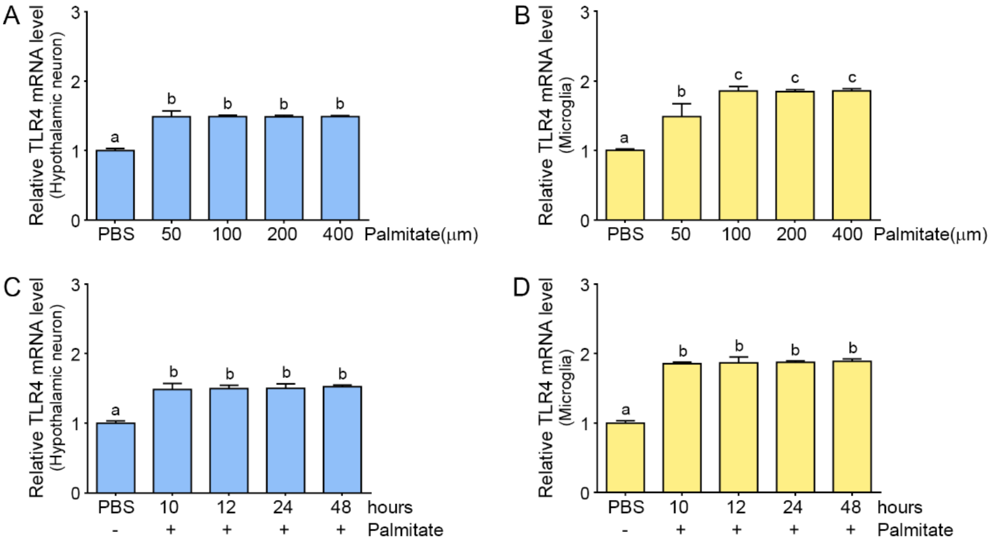

Figure S1. Concentration and duration of palmitate causing ER stress in hypothalamic neurons and microglia TLR4 was confirmed to determine the concentration of palmitate, which causes ER stress due to palmitate. (A) In hypothalamic neurons, TLR4 mRNA level increased to a maximum value at 50 μM palmitate. (B) In microglia, TLR4 expression increased to a maximum value at 100 μM palmitate. (C, D) TLR4 expression began to increase 10 h after the initiation of treatment with palmitate (50 μM in hypothalamic neurons and 100 μM in microglia). Data are means ± SD. Same letters represent no significant difference ($p \geq 0.05$). ER, endoplasmic reticulum; TLR4, Toll-like receptor 4; PBS, phosphate-buffered saline.

Table S1. List of primer for quantitative real time polymerase chain reaction (qRT-PCR)

Gene name		Primer sequence
β-actin	Forward	5'-ACA AAG CTG TTC AGT GTC TCC A-3′
	Reverse	5'-CTC CGT TTC CAG AAT ACA CAC A-3′
TLR4	Forward	5'-ATT CAG AGC CGT TGG TGT ATC T-3′
	Reverse	5'-TCA AGG ACA ATG AAG ATG ATG C-3′
NF-κB	Forward	5'-AGA AAT CCT ACC CAC AGG TCA A-3′
	Reverse	5'-CAT TTG TGA CCA ACT GAA CGA T-3′
PERK	Forward	5'-CAT CAG CAC TTT AGA TGG ACG A-3′
	Reverse	5'-AGA TGA AAC CAA GGA ACC AGA C-3′
eIF2α	Forward	5'-GAA GTG CCT AGT GAG GAG CCT A-3′
	Reverse	5'-CTC TTC ATG CAG TTT GGA ATT G-3′
IRE1	Forward	5'-ATC TGA AAA GGT TCC GCT CAT A-3′
	Reverse	5'-TAG TGG TGC TTC TTG TTC CTC A-3′
Xbp1	Forward	5'-TTG AGG AAG CAC CTC TAA GCT C-3′
	Reverse	5'-GGA TGA AGT CAT CTT CCA AAG G-3′
SOCS3	Forward	5′-ATGGTCACCCACAGCAAGTTT-3′
	Reverse	5′-TCCAGTAGAATCCGCTCTCCT-3′
STAT3	Forward	5'-AGA AAA TGA AGG TGG TGG AGA A-3′
	Reverse	5'-ATT CAG ATC CTG CAT GTC TCC T-3′
ObR	Forward	5′-TGG GCT ACA TCA GGC TTT GAG-3′
	Reverse	5′-CTC TCC TAC AAC CTT CCC CTC-3′
